# Prognostic impact of angiotensin-converting enzyme inhibitors and dexmedetomidine in acute respiratory distress syndrome: a MIMIC-IV-based retrospective cohort analysis

**DOI:** 10.3389/fmed.2025.1601565

**Published:** 2025-08-18

**Authors:** Qingli Hong, Xiaozan Yang, Huahong Yang, Xuanzhao Zhou, Jiaqi Tang, Zhongmei Wen

**Affiliations:** ^1^The First Hospital of Jilin University, Changchun, China; ^2^Changchun Central Hospital Microbiology Laboratory, Changchun, China; ^3^Department of Critical Care Medicine, Guangdong Institute of Cardiovascular Diseases, Guangdong Provincial People’s Hospital, Guangdong Academy of Medical Sciences, Guangzhou, China

**Keywords:** dexmedetomidine, ARDS, MIMIC IV, prognosis, clinical decision

## Abstract

**Background:**

Acute respiratory distress syndrome (ARDS) is a prevalent condition in the respiratory department and intensive care unit that considerably influences prognosis. Prior research has demonstrated that angiotensin-converting enzyme inhibitors (ACEIs) or dexmedetomidine can improve the prognosis of ARDS. Nonetheless, the combinatorial effect of ACEIs and dexmedetomidine on the prognosis of ARDS remains to be investigated.

**Method:**

A retrospective study was conducted using data from 696 patients with ARDS collected from the Medical Information Mart for Intensive Care IV database. Subsequently, a Cox regression model was constructed to screen for meaningful variables. Moreover, a multi-model Cox regression was constructed to exclude the interference of confounding factors and explore the effects of ACEIs alone, dexmedetomidine alone, and ACEIs combined with dexmedetomidine on the prognosis of patients with ARDS. Finally, it was verified by plotting the Kaplan–Meier survival curve.

**Result:**

The survival rates of patients with ARDS within days 28, 60, 90, 180, and 365 after admission were 61.6, 57.0, 55.9, 53.7, and 51.3%, respectively. The results of the multi-model Cox regression revealed that compared with the application of ACEIs or dexmedetomidine alone, ACEIs combined with dexmedetomidine have a synergistic effect on reducing the risk of death in patients with ARDS. The conclusion of the Kaplan–Meier survival curve is consistent with that of the Cox regression.

**Conclusion:**

In terms of reducing the risk of death in patients with ARDS, the combined application of dexmedetomidine and ACEIs may have a better effect than monotherapy.

## 1 Introduction

Acute respiratory distress syndrome (ARDS) is a serious clinical syndrome and a common disease in intensive care units (ICUs). Its pathological features include alveolar–capillary barrier injury, alveolar collapse, ventilator-blood flow ratio imbalance, inflammatory response, oxidative stress imbalance, secondary pulmonary hypertension, and right heart dysfunction (1, 2). The incidence of ARDS is approximately 10.4% (3), and it is associated with high mortality rates in ICUs (4, 5). ARDS is the main cause of respiratory failure and death in patients with ARDS, and its significance is reflected in the high incidence and mortality of ARDS, multiple organ dysfunction, complexity of treatment, and long-term health problems that ARDS survivors may face, such as pulmonary insufficiency, cognitive dysfunction, and decline in quality of life. These long-term effects have had a significant impact on patient health, the medical system, and the social economy (6, 7). Currently, the diagnosis of ARDS is primarily based on the Berlin criteria developed in 2012, which is constantly updated (8, 9). According to these criteria, the in-hospital mortality rate for ARDS is approximately 50% (10). In treating ARDS, the primary goal is to resolve acute respiratory failure. Previous studies have confirmed that angiotensin-converting enzyme inhibitors (ACEIs) can improve the prognosis of outpatients with acute respiratory diseases (11) and reduce the in-hospital mortality rate of patients with acute respiratory failure (12, 13). Additionally, dexmedetomidine has been shown to improve patient prognosis (14–16). There have been relevant studies on the individual application mechanisms and effects of the two drugs on ARDS, which can provide a theoretical basis for potential combined medication. Based on the existing literature search results, no clinical studies have directly explored the combination of ACEIs and dexmedetomidine for ARDS. Currently, the vast majority of clinical studies have focused on the impact of single drugs on the prognosis of patients; however, this does not conform to the actual clinical situation. In clinical practice, it is rare to treat patients with only one drug. Therefore, exploring the impact of combined medication on the prognosis of patients can not only help understand whether there is a synergistic effect of combined medication but also investigate whether the combined application of drugs will exacerbate the side effects of drugs and the probability of complications.

To explore whether there is a synergistic or antagonistic effect between ACEIs and dexmedetomidine on improving the survival rate of patients with ARDS, we aimed to use the Medical Information Mart for Intensive Care (MIMIC IV) database to fill this research gap.

## 2 Methods

### 2.1 Database

This study is based on the MIMIC IV database, which contains information on all patients who received treatment in this institution as well as laboratory records. This information can be used to identify independent risk factors and clinical features of certain diseases (17), thereby assisting clinical decision-making and providing a basis for the formulation of clinical guidelines (18). Diseases were summarized and extracted according to the ninth (ICD-9) and tenth (ICD-10) codes of the International Classification of Diseases (19). One of the authors (Dr. Tang) completed a CITI Data Research course (study ID: 12467279) and obtained access to the MIMIC IV database. In the MIMIC database, since patient information is anonymized, informed consent from patients is not required (20).

### 2.2 Study population

**Figure 1:**
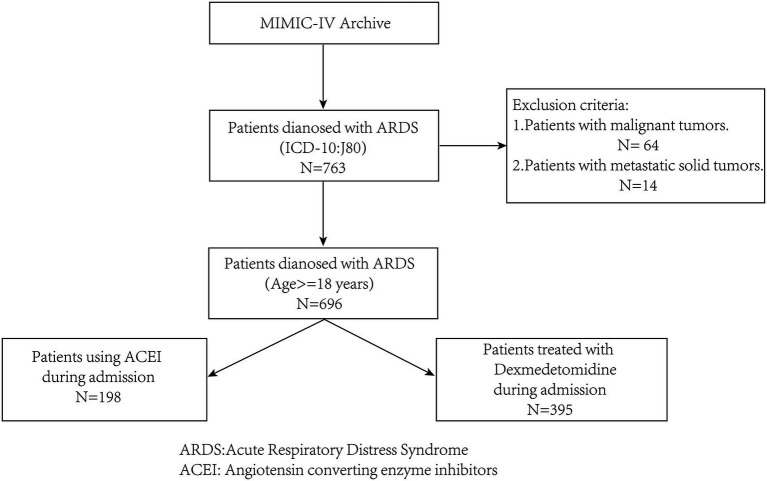
The extraction, cleaning, and screening process of research data.

Patients with ARDS were identified in the database according to the disease code, and patient data were screened according to the following inclusion and exclusion criteria (Figure 1).

#### 2.2.1 Inclusion criteria

The inclusion criteria included: (1) patients with acute respiratory distress syndrome who were admitted to Beth Israel Deaconess Medical Center for the first time, and (2) being at least 18 years old.

#### 2.2.2 Exclusion criteria

The exclusion criteria included: (1) patients with combined malignant and metastatic solid tumors that may significantly impact prognosis, and (2) patients with incomplete information on ACEIs and dexmedetomidine use during hospitalization.

### 2.3 Data processing and analysis

The research data were mainly extracted from Navicat Premium version 16.0, which mainly included the medication information of ACEI and dexmedetomidine, comorbidiasis, and the survival period of patients. The survival period of the patient is calculated from the time of admission. The types of ACEI used in this study include captopril, ramipril, benazepril, quinopril, fosinopril, benazepril, nasepril, perindopril maleate, quinopril, linopril and mosipril.

The cleaned data were imported into R software (version 4.4), and IBM SPSS Statistics (version 25) was used for analysis. First, the Kruskal–Wallis test was performed to compare survival rates across different treatment methods, aiming to evaluate statistical significance (Table 1).

**Table 1:** Significance test of different treatment modalities and prognosis (Kruskal-Wallis Test).

Test Statistics^a^^,^^b^					
Kruskal-Wallis Test	outcome_28d	outcome_60d	outcome_90d	outcome_180d	outcome_365d
Kruskal-Wallis H	130.140	113.754	105.013	97.629	97.410
df	3	3	3	3	3
Asymp. Sig.	<0.001	<0.001	<0.001	<0.001	<0.001

a Kruskal Wallis Test.

b Grouping variable: Cure.

Introdution: According to the Kruskal-Wallis test, the results showed that the *p* value was <0.001 (*p* < 0.05), and it can be considered that the survival rates of patients under the four treatment methods were not all the same.

Cox regression analysis was conducted using the survival package in R. Subsequently, a stepwise Cox regression analysis was conducted. The effects of different treatment methods on the risk of patient death were compared by eliminating interference from covariates. Finally, the R package of survminer was used to plot the Kaplan–Meier survival curve to compare survival rate differences in patients with ARDS under different treatment methods (21).

## 3 Results

### 3.1 Population characteristics

After sorting and cleaning, 696 patients with ARDS were included in this study. Among them, 217 (42.7%) were female. There were 351 (50.4%) patients who were aged 60 years or older. A total of 429, 397, 389, 374, and 357 patients were alive at 28, 60, 90, 180, and 365 days after admission, respectively. On days 28, 60, 90, 180, and 365, survival rates were 61.6, 57.0, 55.9, 53.7, and 51.3%, respectively. There were 198 patients who used ACEIs, 395 patients who used dexmedetomidine, and 113 patients who used ACEIs and dexmedetomidine during admission.

Regarding complications, 98 (14.1%) patients had myocardial infarction. Thirty-five patients (19.4%) had congestive heart failure. Eighty-three patients (11.9%) had cerebrovascular disease. A total of 224 (32.2%) patients were diagnosed with diabetes, accounting for 32.2%. Additionally, 129 patients (18.5%) had kidney disease, and 57 patients had severe liver disease (9.1%).

### 3.2 Cox regression analysis and Kaplan–Meier curve

Multivariate Cox regression analysis showed that treatment methods and severe liver disease were associated with the prognosis of patients with ARDS on days 28, 60, and 90 after admission. Treatment methods, severe liver disease, and kidney disease were associated with outcomes at days 180 and 365 (Tables 2–6).

**Table 2:** Results of univariate and multifactorial cox proportional risk model affecting 28-day survival.

Variables	Univariate				Multivariate			
	*β*	SE	*p*	HR (95%CI)	*β*	SE	*p*	HR (95%CI)
Gender								
1				1.00 (Reference)				1.00 (Reference)
2	0.10	0.12	0.442	1.10 (0.86 ~ 1.40)	−0.01	0.13	0.932	0.99 (0.77 ~ 1.27)
Cure								
0				1.00 (Reference)				1.00 (Reference)
1	−0.60	0.20	0.002	0.55 (0.37 ~ 0.81)	−0.87	0.20	<0.001	0.42 (0.28 ~ 0.62)
2	−1.51	0.15	<0.001	0.22 (0.17 ~ 0.30)	−1.48	0.15	<0.001	0.23 (0.17 ~ 0.31)
3	−2.22	0.26	<0.001	0.11 (0.07 ~ 0.18)	−2.30	0.26	<0.001	0.10 (0.06 ~ 0.17)
Age	0.03	0.00	<0.001	1.03 (1.02 ~ 1.04)	0.03	0.00	<0.001	1.03 (1.02 ~ 1.04)
Chronic pulmonary disease								
0				1.00 (Reference)				1.00 (Reference)
1	0.23	0.14	0.106	1.26 (0.95 ~ 1.66)	0.07	0.15	0.654	1.07 (0.80 ~ 1.42)
Myocardial infarct								
0				1.00 (Reference)				1.00 (Reference)
1	0.36	0.16	0.026	1.44 (1.04 ~ 1.97)	0.20	0.17	0.234	1.23 (0.88 ~ 1.72)
Congestive heart failure								
0				1.00 (Reference)				1.00 (Reference)
1	0.37	0.14	0.010	1.44 (1.09 ~ 1.91)	0.00	0.15	0.988	1.00 (0.74 ~ 1.35)
Cerebrovascular disease								
0				1.00 (Reference)				1.00 (Reference)
1	0.17	0.18	0.331	1.19 (0.84 ~ 1.69)	0.12	0.18	0.513	1.13 (0.79 ~ 1.61)
Diabetes								
0				1.00 (Reference)				1.00 (Reference)
1	0.09	0.13	0.507	1.09 (0.85 ~ 1.41)	0.04	0.14	0.765	1.04 (0.79 ~ 1.38)
Renal disease								
0				1.00 (Reference)				1.00 (Reference)
1	0.38	0.14	0.008	1.46 (1.10 ~ 1.94)	0.25	0.16	0.121	1.28 (0.94 ~ 1.74)
Severe liver disease								
0				1.00 (Reference)				1.00 (Reference)
1	0.69	0.18	<0.001	2.00 (1.40 ~ 2.86)	1.20	0.20	<0.001	3.33 (2.27 ~ 4.89)

HR, Hazard Ratio; CI, Confidence Interval; ACEI, Angiotensin-converting enzyme inhibitor.

Cure: 1-- No ACEI or Dextrometopidine was used during admission. 2--ACEI was used during admission, and Dextrometopidine was not used. 3--No ACEI was used during admission, Dextrometopidine was used. 4-- ACEI and Dextrometopidine were used during admission.

**Table 3:** Results of univariate and multifactorial cox proportional risk model affecting 60-day survival.

Variables	Univariate				Multivariate			
	*β*	SE	*p*	HR (95%CI)	*β*	SE	*p*	HR (95%CI)
Gender								
1				1.00 (Reference)				1.00 (Reference)
2	0.08	0.12	0.506	1.08 (0.86 ~ 1.36)	−0.02	0.12	0.856	0.98 (0.77 ~ 1.24)
Cure								
0				1.00 (Reference)				1.00 (Reference)
1	−0.55	0.19	0.004	0.58 (0.40 ~ 0.83)	−0.82	0.20	<0.001	0.44 (0.30 ~ 0.65)
2	−1.36	0.14	<0.001	0.26 (0.20 ~ 0.34)	−1.34	0.14	<0.001	0.26 (0.20 ~ 0.35)
3	−2.05	0.23	<0.001	0.13 (0.08 ~ 0.20)	−2.14	0.23	<0.001	0.12 (0.07 ~ 0.18)
Age	0.03	0.00	<0.001	1.03 (1.02 ~ 1.04)	0.03	0.00	<0.001	1.03 (1.02 ~ 1.04)
Chronic pulmonary disease								
0				1.00 (Reference)				1.00 (Reference)
1	0.23	0.13	0.090	1.26 (0.97 ~ 1.64)	0.08	0.14	0.573	1.08 (0.82 ~ 1.42)
Myocardial infarct								
0				1.00 (Reference)				1.00 (Reference)
1	0.39	0.15	0.011	1.48 (1.10 ~ 2.00)	0.23	0.16	0.153	1.26 (0.92 ~ 1.73)
Congestive heart failure								
0				1.00 (Reference)				1.00 (Reference)
1	0.33	0.14	0.015	1.40 (1.07 ~ 1.83)	−0.03	0.15	0.814	0.97 (0.72 ~ 1.29)
Cerebrovascular disease								
0				1.00 (Reference)				1.00 (Reference)
1	0.24	0.17	0.152	1.27 (0.92 ~ 1.76)	0.17	0.17	0.308	1.19 (0.85 ~ 1.66)
Diabetes								
0				1.00 (Reference)				1.00 (Reference)
1	0.09	0.12	0.467	1.09 (0.86 ~ 1.39)	0.04	0.14	0.749	1.04 (0.80 ~ 1.36)
Renal disease								
0				1.00 (Reference)				1.00 (Reference)
1	0.42	0.14	0.002	1.52 (1.17 ~ 1.98)	0.29	0.15	0.048	1.34 (1.01 ~ 1.80)
Severe liver disease								
0				1.00 (Reference)				1.00 (Reference)
1	0.76	0.17	<0.001	2.14 (1.53 ~ 2.99)	1.28	0.18	<0.001	3.60 (2.51 ~ 5.15)

HR, Hazard Ratio; CI, Confidence Interval; ACEI, Angiotensin-converting enzyme inhibitor.

Cure:1-- No ACEI or Dextrometopidine was used during admission. 2--ACEI was used during admission, and Dextrometopidine was not used. 3--No ACEI was used during admission, Dextrometopidine was used. 4-- ACEI and Dextrometopidine were used during admission.

**Table 4:** Results of univariate and multifactorial cox proportional risk model affecting 90-day survival.

Variables	Univariate				Multivariate			
	*β*	SE	*p*	HR (95%CI)	*β*	SE	*p*	HR (95%CI)
Gender								
1				1.00 (Reference)				1.00 (Reference)
2	0.06	0.12	0.618	1.06 (0.84 ~ 1.33)	−0.04	0.12	0.741	0.96 (0.76 ~ 1.22)
Cure								
0				1.00 (Reference)				1.00 (Reference)
1	−0.56	0.19	0.003	0.57 (0.39 ~ 0.83)	−0.82	0.19	<0.001	0.44 (0.30 ~ 0.64)
2	−1.32	0.14	<0.001	0.27 (0.20 ~ 0.35)	−1.30	0.14	<0.001	0.27 (0.21 ~ 0.36)
3	−1.94	0.22	<0.001	0.14 (0.09 ~ 0.22)	−2.03	0.22	<0.001	0.13 (0.09 ~ 0.20)
Age	0.03	0.00	<0.001	1.03 (1.02 ~ 1.04)	0.03	0.00	<0.001	1.03 (1.02 ~ 1.04)
Chronic pulmonary disease								
0				1.00 (Reference)				1.00 (Reference)
1	0.21	0.13	0.110	1.24 (0.95 ~ 1.61)	0.07	0.14	0.634	1.07 (0.82 ~ 1.40)
Myocardial infarct								
0				1.00 (Reference)				1.00 (Reference)
1	0.41	0.15	0.007	1.50 (1.12 ~ 2.02)	0.24	0.16	0.133	1.27 (0.93 ~ 1.74)
Congestive heart failure								
0				1.00 (Reference)				1.00 (Reference)
1	0.38	0.13	0.005	1.46 (1.12 ~ 1.89)	0.01	0.14	0.932	1.01 (0.76 ~ 1.34)
Cerebrovascular disease								
0				1.00 (Reference)				1.00 (Reference)
1	0.21	0.17	0.205	1.23 (0.89 ~ 1.71)	0.15	0.17	0.392	1.16 (0.83 ~ 1.61)
Diabetes								
0				1.00 (Reference)				1.00 (Reference)
1	0.10	0.12	0.432	1.10 (0.87 ~ 1.40)	0.04	0.13	0.790	1.04 (0.80 ~ 1.35)
Renal disease								
0				1.00 (Reference)				1.00 (Reference)
1	0.45	0.13	<0.001	1.56 (1.20 ~ 2.03)	0.32	0.15	0.032	1.37 (1.03 ~ 1.83)
Severe liver disease								
0				1.00 (Reference)				1.00 (Reference)
1	0.77	0.17	<0.001	2.16 (1.55 ~ 3.01)	1.29	0.18	<0.001	3.63 (2.55 ~ 5.18)

HR, Hazard Ratio; CI, Confidence Interval; ACEI, Angiotensin-converting enzyme inhibitor.

Cure: 1-- No ACEI or Dextrometopidine was used during admission. 2--ACEI was used during admission, and Dextrometopidine was not used. 3--No ACEI was used during admission, Dextrometopidine was used. 4-- ACEI and Dextrometopidine were used during admission.

**Table 5:** Results of univariate and multifactorial cox proportional risk model affecting 180-day survival.

Variables	Univariate				Multivariate			
	β	SE	*p*	HR (95%CI)	β	SE	*p*	HR (95%CI)
Gender								
1				1.00 (Reference)				1.00 (Reference)
2	0.04	0.11	0.699	1.05 (0.84 ~ 1.31)	−0.05	0.12	0.694	0.95 (0.76 ~ 1.20)
Cure								
0				1.00 (Reference)				1.00 (Reference)
1	−0.45	0.18	0.012	0.64 (0.45 ~ 0.91)	−0.72	0.19	<0.001	0.49 (0.34 ~ 0.70)
2	−1.31	0.13	<0.001	0.27 (0.21 ~ 0.35)	−1.28	0.14	<0.001	0.28 (0.21 ~ 0.36)
3	−1.74	0.20	<0.001	0.17 (0.12 ~ 0.26)	−1.84	0.20	<0.001	0.16 (0.11 ~ 0.23)
Age	0.03	0.00	<0.001	1.03 (1.02 ~ 1.04)	0.03	0.00	<0.001	1.03 (1.02 ~ 1.04)
Chronic pulmonary disease								
0				1.00 (Reference)				1.00 (Reference)
1	0.21	0.13	0.109	1.23 (0.95 ~ 1.59)	0.05	0.13	0.715	1.05 (0.81 ~ 1.37)
Myocardial infarct								
0				1.00 (Reference)				1.00 (Reference)
1	0.43	0.15	0.004	1.53 (1.15 ~ 2.04)	0.23	0.16	0.143	1.25 (0.93 ~ 1.70)
Congestive heart failure								
0				1.00 (Reference)				1.00 (Reference)
1	0.41	0.13	0.002	1.51 (1.17 ~ 1.95)	0.03	0.14	0.835	1.03 (0.78 ~ 1.36)
Cerebrovascular disease								
0				1.00 (Reference)				1.00 (Reference)
1	0.19	0.16	0.248	1.21 (0.88 ~ 1.67)	0.13	0.17	0.431	1.14 (0.82 ~ 1.58)
Diabetes								
0				1.00 (Reference)				1.00 (Reference)
1	0.13	0.12	0.271	1.14 (0.90 ~ 1.44)	0.05	0.13	0.699	1.05 (0.82 ~ 1.36)
Renal disease								
0				1.00 (Reference)				1.00 (Reference)
1	0.50	0.13	<0.001	1.65 (1.28 ~ 2.13)	0.35	0.14	0.013	1.42 (1.08 ~ 1.88)
Severe liver disease								
0				1.00 (Reference)				1.00 (Reference)
1	0.74	0.17	<0.001	2.09 (1.51 ~ 2.91)	1.27	0.18	<0.001	3.57 (2.51 ~ 5.08)

HR, Hazard Ratio; CI, Confidence Interval; ACEI, Angiotensin-converting enzyme inhibitor.

Cure: 1-- No ACEI or Dextrometopidine was used during admission. 2--ACEI was used during admission, and Dextrometopidine was not used. 3--No ACEI was used during admission, Dextrometopidine was used. 4-- ACEI and Dextrometopidine were used during admission.

**Table 6:** Results of univariate and multifactorial cox proportional risk model affecting 365-day survival.

Variables	Univariate				Multivariate			
	*β*	SE	*p*	HR (95%CI)	*β*	SE	*p*	HR (95%CI)
Gender								
1				1.00 (Reference)				1.00 (Reference)
2	0.09	0.11	0.427	1.09 (0.88 ~ 1.36)	0.00	0.11	0.987	1.00 (0.80 ~ 1.25)
Cure								
0				1.00 (Reference)				1.00 (Reference)
1	−0.46	0.18	0.010	0.63 (0.45 ~ 0.90)	−0.73	0.18	<0.001	0.48 (0.34 ~ 0.69)
2	−1.31	0.13	<0.001	0.27 (0.21 ~ 0.35)	−1.27	0.13	<0.001	0.28 (0.22 ~ 0.36)
3	−1.64	0.19	<0.001	0.19 (0.13 ~ 0.28)	−1.74	0.19	<0.001	0.18 (0.12 ~ 0.25)
Age	0.03	0.00	<0.001	1.03 (1.02 ~ 1.04)	0.03	0.00	<0.001	1.03 (1.02 ~ 1.04)
Chronic pulmonary disease								
0				1.00 (Reference)				1.00 (Reference)
1	0.22	0.13	0.088	1.24 (0.97 ~ 1.60)	0.06	0.13	0.667	1.06 (0.82 ~ 1.37)
Myocardial infarct								
0				1.00 (Reference)				1.00 (Reference)
1	0.44	0.14	0.002	1.56 (1.18 ~ 2.06)	0.24	0.15	0.111	1.27 (0.95 ~ 1.71)
Congestive heart failure								
0				1.00 (Reference)				1.00 (Reference)
1	0.43	0.13	<0.001	1.53 (1.19 ~ 1.96)	0.04	0.14	0.768	1.04 (0.80 ~ 1.36)
Cerebrovascular disease								
0				1.00 (Reference)				1.00 (Reference)
1	0.19	0.16	0.228	1.21 (0.89 ~ 1.66)	0.13	0.16	0.440	1.13 (0.82 ~ 1.56)
Diabetes								
0				1.00 (Reference)				1.00 (Reference)
1	0.12	0.12	0.308	1.13 (0.90 ~ 1.41)	0.03	0.13	0.833	1.03 (0.80 ~ 1.32)
Renal disease								
0				1.00 (Reference)				1.00 (Reference)
1	0.50	0.13	<0.001	1.66 (1.29 ~ 2.12)	0.36	0.14	0.011	1.43 (1.09 ~ 1.87)
Severe liver disease								
0				1.00 (Reference)				1.00 (Reference)
1	0.73	0.17	<0.001	2.08 (1.50 ~ 2.88)	1.27	0.18	<0.001	3.56 (2.51 ~ 5.04)

HR, Hazard Ratio; CI, Confidence Interval; ACEI, Angiotensin-converting enzyme inhibitor.

Cure:1-- No ACEI or Dextrometopidine was used during admission. 2--ACEI was used during admission, and Dextrometopidine was not used. 3--No ACEI was used during admission, Dextrometopidine was used. 4-- ACEI and Dextrometopidine were used during admission.

**Figure 2:**
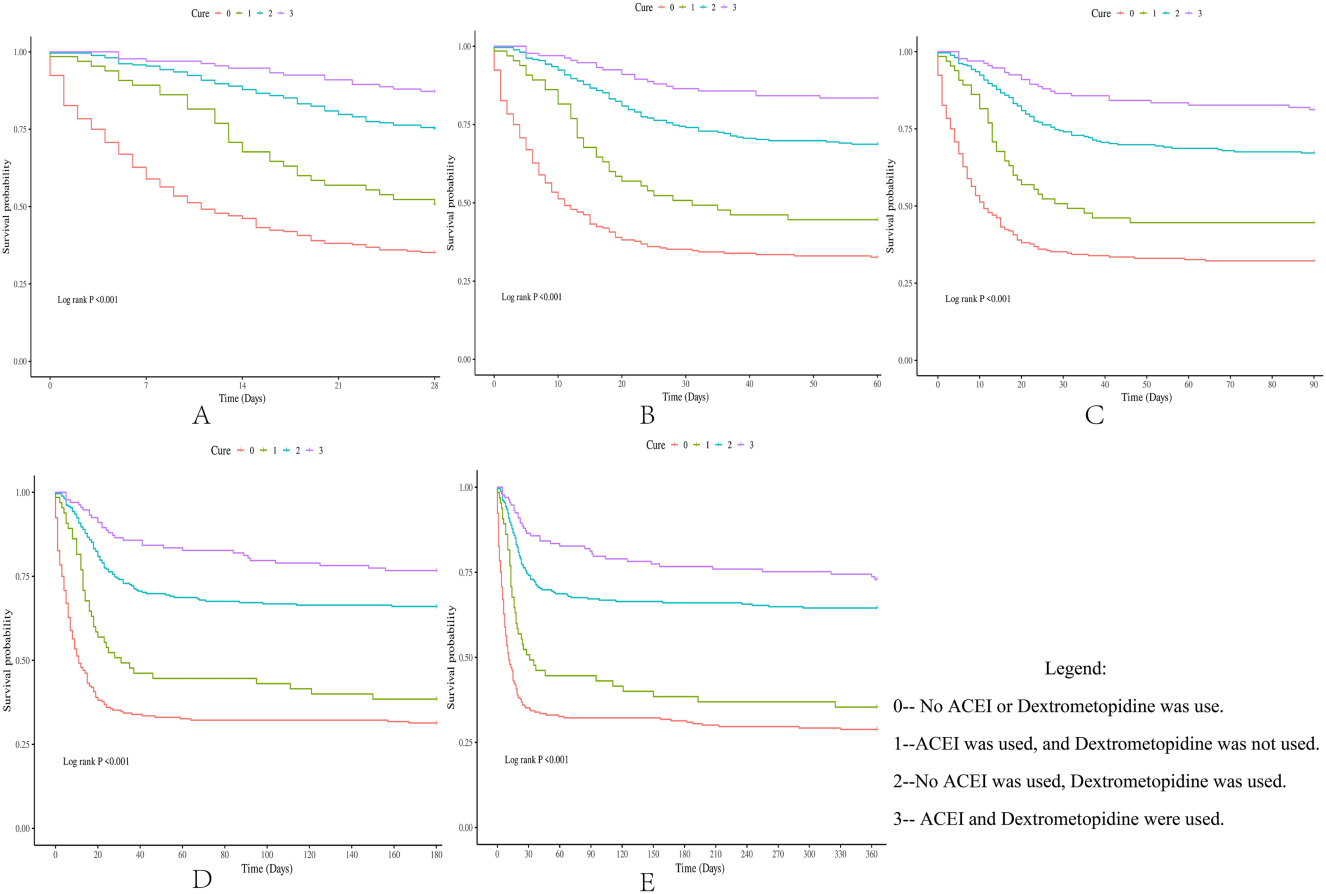
Difference in survival rates between the drug group and the control group (Kaplan-Meier survival curve).

Subsequently, multi-model Cox regression was used to exclude the interference of serious liver disease, kidney disease, and other factors in prognosis, and the adjusted model showed that patients with ARDS who received a combination of ACEIs and dexmedetomidine had the best prognosis. Following treatment with dexmedetomidine alone, patients treated with neither dexmedetomidine nor ACEIs had the worst prognosis. Among them, the risk of death in patients with ARDS treated with ACEIs combined with dexmedetomidine was reduced by 88.7, 86.6, 85.0, 82.4, and 80.6% on days 28, 60, 90, 180, and 365, respectively (Tables 7–11). By drawing the Kaplan–Meier curve, the results showed that, compared to patients treated with ACEI monotherapy or dexmedetomidine monotherapy, the survival rate of patients with ARDS treated with ACEIs and dexmedetomidine was the highest, and the difference was statistically significant (*p* < 0.05) (Figure 2).

**Table 7:** Cox regression model and correction model affecting mortality of ARDS patients within 28 days after admission.

Variables	Model 1		Model 2	
	HR (95%CI)	*p*	HR (95%CI)	*p*
Cure				
0	1.000(Reference)		1.000 (Reference)	
1	0.554 (0.378 ~ 0.811)	0.002	0.413 (0.280 ~ 0.608)	<0.001
2	0.241 (0.180 ~ 0.323)	<0.001	0.249 (0.186 ~ 0.335)	<0.001
3	0.118 (0.071 ~ 0.195)	<0.001	0.113 (0.068 ~ 0.187)	<0.001

HR, Hazard Ratio; CI, Confidence Interval; ACEI, Angiotensin-converting enzymeinhibitor.

Cure: 0-- No ACEI or Dextrometopidine was used during admission. 1--ACEI was used during admission, and Dextrometopidine was not used. 2--No ACEI was used during admission, Dextrometopidine was used. 3-- ACEI and Dextrometopidine were used during admission. Model 1: Crude. Model 2: Adjust: severe_liver_disease, age.

**Table 8:** Cox regression model and correction model affecting mortality of ARDS patients within 60 days after admission.

Variables	Model 1		Model 2	
	HR (95%CI)	*p*	HR (95%CI)	*p*
Cure				
0	1.000 (Reference)		1.000 (Reference)	
1	0.599 (0.417 ~ 0.861)	0.006	0.446 (0.308 ~ 0.645)	<0.001
2	0.284 (0.218 ~ 0.372)	<0.001	0.292 (0.223 ~ 0.382)	<0.001
3	0.140 (0.090 ~ 0.219)	<0.001	0.134 (0.086 ~ 0.210)	<0.001

HR, Hazard Ratio; CI, Confidence Interval.

Cure: 0-- No ACEI or Dextrometopidine was used during admission. 1--ACEI was used during admission, and Dextrometopidine was not used. 2--No ACEI was used during admission, Dextrometopidine was used. 3-- ACEI and Dextrometopidine were used during admission. Model 1: Crude. Model 2: Adjust: severe_liver_disease, age.

**Table 9:** Cox regression model and correction model affecting mortality of ARDS patients within 90 days after admission.

Variables	Model 1		Model 2	
	HR (95%CI)	*p*	HR (95%CI)	*p*
Cure				
0	1.000 (Reference)		1.000 (Reference)	
1	0.596 (0.415 ~ 0.855)	0.005	0.446 (0.308 ~ 0.644)	<0.001
2	0.295 (0.226 ~ 0.384)	<0.001	0.302 (0.232 ~ 0.394)	<0.001
3	0.157 (0.103 ~ 0.239)	<0.001	0.150 (0.098 ~ 0.229)	<0.001

HR: Hazard Ratio, CI: Confidence Interval. ACEI: Angiotensin-converting enzymeinhibitor.

Cure: 0-- No ACEI or Dextrometopidine was used during admission. 1--ACEI was used during admission, and Dextrometopidine was not used. 2--No ACEI was used during admission, Dextrometopidine was used. 3-- ACEI and Dextrometopidine were used during admission. Model 1: Crude. Model 2: Adjust: severe_liver_disease, age.

**Table 10:** Cox regression model and correction model affecting mortality of ARDS patients within 180 days after admission.

Variables	Model 1		Model 2	
	HR (95%CI)	*p*	HR (95%CI)	*p*
Cure				
0	1.000 (Reference)		1.000 (Reference)	
1	0.656 (0.464 ~ 0.927)	0.017	0.506 (0.355 ~ 0.720)	<0.001
2	0.298 (0.230 ~ 0.387)	<0.001	0.308 (0.237 ~ 0.400)	<0.001
3	0.189 (0.129 ~ 0.278)	<0.001	0.176 (0.119 ~ 0.259)	<0.001

HR, Hazard Ratio; CI, Confidence Interval; ACEI, Angiotensin-converting enzymeinhibitor.

Cure: 0-- No ACEI or Dextrometopidine was used during admission. 1--ACEI was used during admission, and Dextrometopidine was not used. 2--No ACEI was used during admission, Dextrometopidine was used. 3-- ACEI and Dextrometopidine were used during admission. Model 1: Crude. Model 2: Adjust: renal_disease, severe_liver_disease, age.

**Table 11:** Cox regression model and correction model affecting mortality of ARDS patients within 365 days after admission.

Variables	Model1		Model 2	
	HR (95%CI)	*p*	HR (95%CI)	*p*
Cure				
0	1.000 (Reference)		1.000 (Reference)	
1	0.667 (0.475 ~ 0.935)	0.019	0.510 (0.361 ~ 0.720)	<0.001
2	0.296 (0.230 ~ 0.382)	<0.001	0.308 (0.238 ~ 0.398)	<0.001
3	0.208 (0.145 ~ 0.299)	<0.001	0.194 (0.135 ~ 0.279)	<0.001

HR, Hazard Ratio; CI:, Confidence Interval; ACEI, Angiotensin-converting enzymeinhibitor.

Cure: 0-- No ACEI or Dextrometopidine was used during admission. 1--ACEI was used during admission, and Dextrometopidine was not used. 2--No ACEI was used during admission, Dextrometopidine was used. 3-- ACEI and Dextrometopidine were used during admission. Model 1: Crude. Model 2: Adjust: renal_disease, severe_liver_disease, age.

## 4 Discussion

ARDS is characterized by diffuse pulmonary interstitial and alveolar edema caused by injury to pulmonary capillary endothelial cells and alveolar epithelial cells induced by non-cardiogenic diseases, such as trauma and severe infection, followed by acute hypoxic respiratory insufficiency (22–27). Current research indicates that ARDS can be divided into two distinct subphenotypes. One subphenotype is characterized by more severe inflammation, shock, and metabolic acidosis, and a poorer clinical prognosis. In a randomized trial examining PEEP strategies, these subtypes responded differently to treatment (28). Current treatment methods include improved ventilation (29–32), respiratory support (33, 34), sedation (35), muscle relaxation (36), and early nerve block (37–39).

Previous studies have shown that both ACEI and dexmedetomidine monotherapies benefit patients with ARDS. However, whether the combined application of ACEIs and dexmedetomidine can play a synergistic role in improving the survival rate of patients with ARDS remains to be explored. Four treatment methods were established in this study: neither ACEIs nor dexmedetomidine was used; ACEIs were used instead of dexmedetomidine; dexmedetomidine was used instead of ACEIs; and ACEIs and dexmedetomidine were used. By comparing the survival rate differences of patients with ARDS under four different treatment methods, this study explored whether the combined application of ACEIs and dexmedetomidine could further enhance the improvement in the prognosis of patients with ARDS. By constructing a multi-model Cox regression coarse model, the results showed that compared to the other three treatment methods, the combined application of ACEIs and dexmedetomidine had the strongest ability to reduce the risk of death in patients with ARDS. Subsequently, by adjusting for the interference of covariates, the adjusted model showed that, compared with the other three groups, patients with ARDS who received ACEIs and dexmedetomidine had the highest survival rate, and this conclusion was maintained within 0–365 days after admission. Finally, through the Kaplan–Meier survival curve analysis in this study, it was further verified that the combined application of ACEIs and dexmedetomidine had a synergistic effect on improving the prognosis of patients with ARDS.

As the combined application of dexmedetomidine and ACEIs has not yet been reported, it is unfeasible to draw conclusions from previous studies to explain our results. Therefore, we can only explore why ACEIs combined with dexmedetomidine significantly improve the survival rate of patients with ARDS from the perspective of the mechanism of drug action.

ACEIs are a class of drugs that reduce the production of angiotensin II by inhibiting angiotensin-converting enzymes, which dilate blood vessels and reduce peripheral resistance (40, 41). In addition, ACEIs may have anti-inflammatory effects because angiotensin II is thought to be involved in pro-inflammatory responses (42, 43). In ARDS, an excessive inflammatory response in the lungs may be an important factor leading to lung injury (24). Therefore, inhibiting angiotensin II synthesis may help reduce inflammation and improve lung function (44, 45). Dexmedetomidine is a selective α2 adrenergic receptor agonist that promotes sodium and water excretion. As ARDS is often associated with fluid retention and pulmonary edema, the use of dexmedetomidine may also help improve fluid balance, indirectly reduce pulmonary edema, and improve oxygenation. In addition, dexmedetomidine maintained hemodynamic stability (46). Finally, dexmedetomidine neither leads to deeper sedation levels nor does it lead to respiratory depression, which is conducive to rapid patient recovery (47, 48).

Based on the above drug mechanism analysis, we found that the combination of ACEIs and dexmedetomidine has certain potential advantages. For example, the anti-inflammatory effects of ACEIs and the sedative and protective effects of dexmedetomidine may synergistically improve lung function and oxygenation. The combination of these may intervene in the pathophysiological process of ARDS through multiple targets, reduce the mortality rate, and shorten the duration of mechanical ventilation. In addition, ACEIs can inhibit ROS generation, and dexmedetomidine activates the Nrf2 pathway. This combined application may enhance the antioxidant capacity of alveolar epithelial cells and reduce apoptosis.

Although we tried our best to improve the research plan, there were some limitations. First, this was a single-center, retrospective study. The strength of the conclusions drawn from this retrospective study is not as strong as that of prospective studies. Therefore, in the future, multicenter prospective studies need to be improved for verification. Second, this study preliminarily explored and verified the improvement effect of ACEIs and dexmedetomidine on the prognosis of patients with ARDS based on statistics and made predictions about the conclusion in combination with the mechanisms of the two types of drugs. The basic experiments need to be improved in the future to further verify these speculations. Finally, because of the limitations of the database, other confounding factors, such as smoking history, may not have been included in this study. Moreover, there has been no further study on the correlation between drug exposure time and the prognosis of patients with ARDS. Despite these limitations, this study provides novel insights for future prospective research and basic experiments.

## 5 Conclusion

ARDS is a complication with acute onset and poor prognosis, and its treatment remains complex. However, significant progress has been made in the treatment of ARDS. For example, dexmedetomidine has been shown to improve patient prognosis. Prone positioning has improved ventilation. Despite these advances, consensus has not yet been reached on some issues. For example, while ACEIs can improve the prognosis of patients with ARDS, it is still unclear whether combining dexmedetomidine with ACEIs is superior to using dexmedetomidine alone.

In this study, the improvement effect of ACEIs on the prognosis of patients with ARDS was confirmed, the effect of dexmedetomidine was verified, and it was further found that the combined application of dexmedetomidine and ACEIs has a certain synergistic effect on the improvement of the prognosis of patients with ARDS. In the future, it will be necessary to conduct prospective studies to verify the conclusions of this study and improve basic research to explore the underlying molecular mechanisms.

## Data Availability

The original contributions presented in the study are included in the article/supplementary material, further inquiries can be directed to the corresponding author.

## References

[1] Umbrello M Fumagalli J Pesenti A Chiumello D. Pathophysiology and Management of Acute Respiratory Distress Syndrome in obese patients. Semin Respir Crit Care Med. (2019) 40:40–56. doi: 10.1055/s-0039-1685179, PMID: 31060087

[2] Gattinoni L Pelosi P Suter PM Pedoto A Vercesi P Lissoni A. Acute respiratory distress syndrome caused by pulmonary and extrapulmonary disease. Different syndromes? Am J Respir Crit Care Med. (1998) 158:3–11. doi: 10.1164/ajrccm.158.1.9708031, PMID: 9655699

[3] Bellani G Laffey JG Pham T Fan E Brochard L Esteban A. Epidemiology, patterns of care, and mortality for patients with acute respiratory distress syndrome in intensive care units in 50 countries. JAMA. (2016) 315:788–800. doi: 10.1001/jama.2016.0291 26903337

[4] Acute Respiratory Distress Syndrome Network Brower RG Matthay MA Morris A Schoenfeld D Thompson BT. Ventilation with lower tidal volumes as compared with traditional tidal volumes for acute lung injury and the acute respiratory distress syndrome. N Engl J Med. (2000) 342:1301–8. doi: 10.1056/NEJM200005043421801, 10793162

[5] Zambon M Vincent JL. Mortality rates for patients with acute lung injury/ARDS have decreased over time. Chest. (2008) 133:1120–7. doi: 10.1378/chest.07-2134, PMID: 18263687

[6] Ohshimo S. Oxygen administration for patients with ARDS. J Intensive Care. (2021) 9:17. doi: 10.1186/s40560-021-00532-0, PMID: 33549131 PMC7865109

[7] Kimura S Stoicea N Rosero Britton BR Shabsigh M Branstiter A Stahl DL. Preventing ventilator-associated lung injury: a perioperative perspective. Front Med (Lausanne). (2016) 3:25. doi: 10.3389/fmed.2016.00025, PMID: 27303668 PMC4885020

[8] Thompson BT Moss M. A new definition for the acute respiratory distress syndrome. Semin Respir Crit Care Med. (2013) 34:441–7. doi: 10.1055/s-0033-1351162, PMID: 23934713

[9] Matthay MA Arabi Y Arroliga AC Bernard G Bersten AD Brochard LJ. A new global definition of acute respiratory distress syndrome. Am J Respir Crit Care Med. (2024) 209:37–47. doi: 10.1164/rccm.202303-0558WS, PMID: 37487152 PMC10870872

[10] Papazian L Pauly V Hamouda I Daviet F Orleans V Forel JM. National incidence rate and related mortality for acute respiratory distress syndrome in France. Anaesth Crit Care Pain Med. (2021) 40:100795. doi: 10.1016/j.accpm.2020.100795, PMID: 33359625 PMC9896966

[11] Jeffery MM Cummins NW Dempsey TM Limper AH Shah ND Bellolio F. Association of outpatient ACE inhibitors and angiotensin receptor blockers and outcomes of acute respiratory illness: a retrospective cohort study. BMJ Open. (2021) 11:e044010. doi: 10.1136/bmjopen-2020-044010, PMID: 33737435 PMC7978099

[12] Fang YP Zhang X. A propensity score-matching analysis of angiotensin-converting enzyme inhibitor and angiotensin receptor blocker exposure on in-hospital mortality in patients with acute respiratory failure. Pharmacotherapy. (2022) 42:387–96. doi: 10.1002/phar.2677, PMID: 35344607 PMC9322533

[13] Akyüz A Işık F Aslan B Çap M Kaya İ Atlı Ö. The effect of RAAS inhibitors on acute hypoxemic respiratory failure and in-hospital mortality in the hypertensive Covid-19 patients. Clin Exp Hypertens. (2021) 43:587–96. doi: 10.1080/10641963.2021.1916947, PMID: 33955313 PMC8108186

[14] Song Y Gao S Tan W Qiu Z Zhou H Zhao Y. Dexmedetomidine versus midazolam and propofol for sedation in critically ill patients: mining the medical information Mart for intensive care data. Ann Transl Med. (2019) 7:197. doi: 10.21037/atm.2019.04.14, PMID: 31205915 PMC6545304

[15] Boncyk C Devlin JW Faisal H Girard TD Hsu SH Jabaley CS. INhaled sedation versus Propofol in REspiratory failure in the intensive care unit (INSPiRE-ICU1): protocol for a randomised, controlled trial. BMJ Open. (2024) 14:e086946. doi: 10.1136/bmjopen-2024-086946, PMID: 39461861 PMC11529737

[16] Shi X Zhang J Sun Y Chen M Han F. Effect of different sedatives on the prognosis of patients with mechanical ventilation: a retrospective cohort study based on MIMIC-IV database. Front Pharmacol. (2024) 15:1301451. doi: 10.3389/fphar.2024.1301451, PMID: 39092229 PMC11291308

[17] Zhang Z Goyal H Lange T Hong Y. Healthcare processes of laboratory tests for the prediction of mortality in the intensive care unit: a retrospective study based on electronic healthcare records in the USA. BMJ Open. (2019) 9:e028101. doi: 10.1136/bmjopen-2018-028101, PMID: 31239303 PMC6597637

[18] Kurniati AP Rojas E Hogg D Hall G Johnson OA. The assessment of data quality issues for process mining in healthcare using medical information Mart for intensive care III, a freely available e-health record database. Health Informatics J. (2019) 25:1878–93. doi: 10.1177/1460458218810760, PMID: 30488750

[19] Johnson AE Bulgarelli L Shen L Gayles A Shammout A Horng S. MIMIC-IV, a freely accessible electronic health record dataset. Sci Data. (2023) 10:1. doi: 10.1038/s41597-022-01899-x 36596836 PMC9810617

[20] Cusack R Garduno A Elkholy K Martín-Loeches I. Novel investigational treatments for ventilator-associated pneumonia and critically ill patients in the intensive care unit. Expert Opin Investig Drugs. (2022) 31:173–92. doi: 10.1080/13543784.2022.2030312, PMID: 35040388

[21] Gomes AP Costa B Marques R Nunes V Coelho C. Kaplan-Meier survival analysis: practical insights for clinicians. Acta Medica Port. (2024) 37:280–5. doi: 10.20344/amp.21080, PMID: 38631048

[22] Matthay MA Zemans RL Zimmerman GA Arabi YM Beitler JR Mercat A. Acute respiratory distress syndrome. Nat Rev Dis Primers. (2019) 5:18. doi: 10.1038/s41572-019-0069-0, PMID: 30872586 PMC6709677

[23] Bossardi Ramos R Adam AP. Molecular mechanisms of vascular damage during lung injury. Adv Exp Med Biol. (2021) 1304:95–107. doi: 10.1007/978-3-030-68748-9_6, PMID: 34019265 PMC8223730

[24] Bernard GR Artigas A Brigham KL Carlet J Falke K Hudson L. The American-European consensus conference on ARDS. Definitions, mechanisms, relevant outcomes, and clinical trial coordination. Am J Respir Crit Care Med. (1994) 149:818–24. doi: 10.1164/ajrccm.149.3.7509706, PMID: 7509706

[25] Schmidt M Hajage D Lebreton G Dres M Guervilly C Richard JC. Prone positioning during extracorporeal membrane oxygenation in patients with severe ARDS: the PRONECMO randomized clinical trial. JAMA. (2024) 331:446. doi: 10.1001/jama.2023.24491 38038395 PMC10692949

[26] Guérin C Reignier J Richard JC Beuret P Gacouin A Boulain T. Prone positioning in severe acute respiratory distress syndrome. N Engl J Med. (2013) 368:2159–68. doi: 10.1056/NEJMoa1214103, PMID: 23688302

[27] Scholten EL Beitler JR Prisk GK Malhotra A. Treatment of ARDS with prone positioning. Chest. (2017) 151:215–24. doi: 10.1016/j.chest.2016.06.032, PMID: 27400909 PMC6026253

[28] Calfee CS Delucchi K Parsons PE Thompson BT Ware LB Matthay MA. Subphenotypes in acute respiratory distress syndrome: latent class analysis of data from two randomised controlled trials. Lancet Respir Med. (2014) 2:611–20. doi: 10.1016/S2213-2600(14)70097-9, PMID: 24853585 PMC4154544

[29] Ashra F Chen R Kang XL Chiang KJ Pien LC Jen HJ. Effectiveness of prone position in acute respiratory distress syndrome and moderating factors of obesity class and treatment durations for COVID-19 patients: a meta-analysis. Intensive Crit Care Nurs. (2022) 72:103257. doi: 10.1016/j.iccn.2022.103257, PMID: 35672215 PMC8995327

[30] Subgroup of Critical Respiratory Diseases. Standardized protocol of prone position ventilation in patients with acute respiratory distress syndrome. Zhonghua Nei Ke Za Zhi. (2020) 59:781–7. doi: 10.3760/cma.j.cn112138-20200430-00439 32987480

[31] Camporota L Sanderson B Chiumello D Terzi N Argaud L Rimmelé T. Prone position in COVID-19 and -COVID-19 acute respiratory distress syndrome: an international multicenter observational comparative study. Crit Care Med. (2022) 50:633–43. doi: 10.1097/CCM.0000000000005354, PMID: 34582426 PMC8923275

[32] Besch G Persoz MO Liaudet L. Décubitus ventral et syndrome de détresse respiratoire aiguë de l'adulte: de la théorie à la pratique [Prone position mechanical ventilation in the acute respiratory distress syndrome: theoretical and practical considerations]. Rev Med Suisse. (2014) 10:2362–7. doi: 10.1056/NEJMoa1901686 25632631

[33] Umbrello M Marino A Chiumello D. Tidal volume in acute respiratory distress syndrome: how best to select it. Ann Transl Med. (2017) 5:287. doi: 10.21037/atm.2017.06.51, PMID: 28828362 PMC5537119

[34] Beitler JR Sarge T Banner-Goodspeed VM Gong MN Cook D Novack V. Effect of titrating positive end-expiratory pressure (PEEP) with an esophageal pressure-guided strategy vs an empirical high PEEP-Fio2 strategy on death and days free from mechanical ventilation among patients with acute respiratory distress syndrome: a randomized clinical trial. JAMA. (2019) 321:846–57. doi: 10.1001/jama.2019.0555, PMID: 30776290 PMC6439595

[35] Jabaudon M Boucher P Imhoff E Chabanne R Faure JS Roszyk L. Sevoflurane for sedation in acute respiratory distress syndrome. A randomized controlled pilot study. Am J Respir Crit Care Med. (2017) 195:792–800. doi: 10.1164/rccm.201604-0686OC, PMID: 27611637

[36] Moore L Kramer CJ Delcoix-Lopes S Modrykamien AM. Comparison of Cisatracurium versus Atracurium in early ARDS. Respir Care. (2017) 62:947–52. doi: 10.4187/respcare.05102, PMID: 28351905

[37] National Heart, Lung, and Blood Institute PETAL Clinical Trials Network Moss M Huang DT Brower RG Ferguson ND Ginde AA. Early neuromuscular blockade in the acute respiratory distress syndrome. N Engl J Med. (2019) 380:1997–2008. 31112383 10.1056/NEJMoa1901686 PMC6741345

[38] Grawe ES Bennett S Hurford WE. Early paralysis for the management of ARDS. Respir Care. (2016) 61:830–8. doi: 10.4187/respcare.04734, PMID: 27094392

[39] Bourenne J Hraiech S Roch A Gainnier M Papazian L Forel JM. Sedation and neuromuscular blocking agents in acute respiratory distress syndrome. Ann Transl Med. (2017) 5:291. doi: 10.21037/atm.2017.07.19, PMID: 28828366 PMC5537113

[40] Kellner M Noonepalle S Lu Q Srivastava A Zemskov E Black SM. ROS signaling in the pathogenesis of acute lung injury (ALI) and acute respiratory distress syndrome (ARDS). Adv Exp Med Biol. (2017) 967:105–37. doi: 10.1007/978-3-319-63245-2_8, PMID: 29047084 PMC7120947

[41] Mittal M Siddiqui MR Tran K Reddy SP Malik AB. Reactive oxygen species in inflammation and tissue injury. Antioxid Redox Signal. (2014) 20:1126–67. doi: 10.1089/ars.2012.5149, PMID: 23991888 PMC3929010

[42] Nguyen Dinh Cat A Montezano AC Burger D Touyz RM. Angiotensin II, NADPH oxidase, and redox signaling in the vasculature. Antioxid Redox Signal. (2013) 19:1110–20. doi: 10.1089/ars.2012.4641, PMID: 22530599 PMC3771549

[43] Zheng J Li G Chen S Bihl J Buck J Zhu Y. Activation of the ACE2/Ang-(1-7)/mas pathway reduces oxygen-glucose deprivation-induced tissue swelling, ROS production, and cell death in mouse brain with angiotensin II overproduction. Neuroscience. (2014) 273:39–51. doi: 10.1016/j.neuroscience.2014.04.060, PMID: 24814023 PMC4159741

[44] Ekström M Bornefalk-Hermansson A. Cardiovascular and antacid treatment and mortality in oxygen-dependent pulmonary fibrosis: a population-based longitudinal study. Respirology. (2016) 21:705–11. doi: 10.1111/resp.12781, PMID: 27009834

[45] Kriszta G Kriszta Z Váncsa S Hegyi PJ Frim L Erőss B. Effects of angiotensin-converting enzyme inhibitors and angiotensin receptor blockers on angiotensin-converting enzyme 2 levels: a comprehensive analysis based on animal studies. Front Pharmacol. (2021) 12:619524. doi: 10.3389/fphar.2021.619524, PMID: 33762942 PMC7982393

[46] McKenzie-Brown AM. Commentary on: total intravenous anesthesia with dexmedetomidine for hemodynamic stability and enhanced recovery in facial aesthetic surgery. Aesthet Surg J. (2022) 42:NP611–2. doi: 10.1093/asj/sjac183, PMID: 35788825

[47] Lewis K Piticaru J Chaudhuri D Basmaji J Fan E Møller MH. Safety and efficacy of Dexmedetomidine in acutely ill adults requiring noninvasive ventilation: a systematic review and meta-analysis of randomized trials. Chest. (2021) 159:2274–88. doi: 10.1016/j.chest.2020.12.052, PMID: 33434496 PMC8579314

[48] Chanques G Constantin JM Devlin JW Ely EW Fraser GL Gélinas C. Analgesia and sedation in patients with ARDS. Intensive Care Med. (2020) 46:2342–56. doi: 10.1007/s00134-020-06307-9, PMID: 33170331 PMC7653978

